# LONGITUDINAL ASSOCIATION BETWEEN SERUM 25 HYDROXY VITAMIN D AND COGNITIVE FUNCTION AMONG ICELANDIC OLDER ADULTS

**DOI:** 10.1093/geroni/igz038.1799

**Published:** 2019-11-08

**Authors:** Hrafnhildur Eymundsdottir, Milan Chang, Olof Geirsdottir, Maria Jonsdottir, Palmi V Jonsson, Vilmundur Gudnason, Alfons Ramel

**Affiliations:** 1 The Icelandic Gerontological Research Institute, Reykjavik, Iceland; 2 University of Iceland, School of Education, Faculty of Health Promotion, Sports and Leisure Studies, Reykjavik, Iceland; 3 Reykjavik University, Reykjavik, Iceland; 4 Landspitali University Hospital, Reykjavik, Iceland; 5 Icelandic Heart Association, Kopavogur, Iceland; 6 University of Iceland, Reykjavik, Capital area, Iceland

## Abstract

Studies have indicated that low levels of serum 25 hydroxy vitamin D (25OHD) are associated with lower cognitive function among older adults while longitudinal studies have revealed controversial results. The aim was to investigate the longitudinal associations between 25OHD and cognitive function among older adults with 5-years follow up. The Age, Gene/Environment Susceptibility (AGES)-Reykjavik Study (N=3411) assessed cognitive function measuring memory function, speed of processing and executive function. 25OHD was measured using the Liaison chemiluminescence immunoassay and used as a continuous variable. Multivariate linear analysis, adjusting for numerous confounding factors, was used to calculate the longitudinal associations. All analyses were performed separated by gender. There was a high tendency for low levels of 25OHD i.e. 29.6% men and 37.7% women had hypovitaminosis D (<50 nmol/l). Both men and women had significantly lower scores in all aspects of cognitive function at the follow-up time period. Unadjusted correlations between 25OHD and cognitive functions showed a stronger correlation for women, whereas women had lower scores in all aspects of cognitive function associated with low 25OHD. After adjusting for potential confounders, e.g. age, education, lifestyle and health-related factors, 25OHD and cognitive function were not significantly associated. Observational studies indicate that lower levels of vitamin D are associated with lower cognitive function. Intervention studies are yet to show a clear benefit from vitamin D supplementation. More longitudinal- and interventional studies, with longer follow-up duration, are needed.

